# Potential therapeutic approach of intravenous immunoglobulin against COVID-19

**DOI:** 10.1186/s13223-021-00609-3

**Published:** 2021-10-09

**Authors:** Zahra Kolahchi, Hanye Sohrabi, Sara Ekrami Nasab, Hesan Jelodari Mamaghani, Maryam Keyfari Alamdari, Nima Rezaei

**Affiliations:** 1 Students’ International Committee of Medical Schools (SICoMS), School of Medicine, Tehran University of Medical Sciences, Tehran, Iran; 2 Research Center for Immunodeficiencies, Children’s Medical Center, Tehran University of Medical Sciences, Dr. Qarib St, Keshavarz Blvd, 14194 Tehran, Iran; 3 Department of Immunology, School of Medicine, Tehran University of Medical Sciences, Tehran, Iran; 4 Network of Immunity in Infection, Malignancy and Autoimmunity (NIIMA), Universal Scientific Education and Research Network (USERN), Tehran, Iran

**Keywords:** Coronavirus disease, Intravenous immunoglobulin, Respiratory syndromes, Hyperreactivity, Treatment

## Abstract

Since the outbreak of the novel coronavirus disease (COVID-19), the therapeutic and management options to reduce the burden of the COVID-19 disease are under investigation. IVIG therapy is used as an effective treatment for immunodeficient patients and patients with inflammatory or autoimmune conditions. The therapeutic effect of IVIG in COVID-19 patients has been investigated. But, the results are controversial and some studies reported no benefit of IVIG therapy. More clinical trials on the effect of IVIG therapy in COVID-19 patients should be performed to establish a certain conclusion about IVIG effectiveness.

## Introduction

In late 2019, a new type of Coronavirus, severe acute respiratory syndrome coronavirus 2 (SARS-CoV-2), was introduced and soon gave rise to a world-level pandemic [1]. As of today, the case fatality ratio (CFR) of COVID-19 ranges from less than 0.1% to more than 25% varying between countries, and the overall CFR is probably around 1% [2, 3]. This led to a global lockdown and detailed study of the disease and attempts to provide a treatment or vaccine [4].

A major pathophysiological finding in relatively more severe COVID-19 patients has been hyper inflammation, which will be targeted in this study. Elevated levels of interleukin (IL)-2, IL-6, IL-7, interferon-gamma (IFN-γ) inducible protein 10 (IP-10), and tumor necrosis factor-alpha (TNF-α) indicates a cytokine cascade and a cytokine release syndrome as an underlying immunopathology [5, 6]. Furthermore, the elevation of C-reactive protein (CRP), lactate dehydrogenase (LDH), D-dimer, and ferritin in infected patients, showed more evidence of a hyperinflammatory state [7, 8]. This systemic inflammation gives rise to the infiltration of monocytes and lymphocytes in the lung and the heart. Particularly, pathogenic granulocyte–macrophage colony-stimulating factor (GM-CSF)-secreting T-cells were shown to correlate with the recruitment of inflammatory IL-6-secreting monocytes and severe lung pathology in COVID-19 patients [9].

In the 1960s, a new method of treatment for inflammation-related diseases was introduced. [10] Immunoglobulin therapy (IVIG) is based on the intravenous injection of human immunoglobulin to patients suffering from specific inflammatory conditions. IVIG is a pool of collected antibodies (IgG types) from healthy donors [11]. Generally, plasma of multiple healthy donors was used to extract immunoglobulin for IVIG therapy; nevertheless, it has been suggested that plasma of the people who have recovered from severe viral infections may be more effective [12]. We performed this review to summarize recent studies and knowledge about the effectiveness of IVIG treatment for COVID-19**.**

## IVIG: definition, structure, and role in the treatment

IVIG is routinely used as an effective treatment for primary immunodeficiency, immune thrombocytopenic purpura, Kawasaki disease, dermatomyositis, and Guillain-Barré syndrome primarily as an antibody moderator [13]. IVIG is administered in two distinct types of conditions with specific doses. First, it can be used as replacement therapy with a peak serum level of 12–14 mg/ml for immunodeficient patients. Second, administering a high dose of IVIG to reach a serum level of 25–35 mg/ml can be used in patients with inflammatory or autoimmune conditions. IVIG therapy generally functions as a replacement for the missing antibodies in the first type of administration (replacement therapy). However, it has other roles, such as normalizing monocyte differentiation toward DC [14] and inducing proliferation of B cells and antibody production in CVID patients[15].

Moreover, IVIG therapy has multi-level effects on the immune system in the second type (high-dose IVIG). It has been shown that the positive effects of the IVIG therapy remain far beyond the IgG half-life, which extends IVIG function from a simple injection of antibodies to a more stable and effective method of treatment [14]. Furthermore, IVIG can modulate the immune response by suppressing the Th17 response and expanding regulatory T-cells through induction of cyclo-oxygenase-2-dependent prostaglandin E2 production in dendritic cells. Another proposed mechanism is the blockage of Fas-mediated cell death, causing a decrease in inflammatory reactions [16, 17]. A more recent study has shown that immunoglobulins can decrease the ability of activated T cells in engaging with microglia, a mechanism that led to the detection of lower levels of tumor necrosis factor-alpha and interleukin-10 in T-cell- microglia co-culture [18].

It was found that continuous injection of IVIG influences the process of lymphocyte differentiation and maturation, which finally leads to the normal immune response of white blood cells and prevents the production of inflammatory factors. Therefore, it decreases the injury caused by inflammation [19–21].

## Previous experiences of using IVIG for respiratory syndromes

Several studies have reported the results of IVIG therapy in different respiratory syndromes, including severe acute respiratory syndrome (SARS), Middle East respiratory syndrome (MERS), acute respiratory distress syndrome (ARDS), influenza pandemics, and seasonal influenza [22, 23]. There is no adequate evidence to confirm that using IVIG is beneficial for SARS patients [24, 25], however, there are controversies about using IVIG against MERS [26, 27].

As an adjunct treatment in severe influenza, IVIG is an efficacious treatment for the pulmonary lesions in influenza pneumonia and *Mycoplasma pneumoniae* pneumonia [28–30]. Prevention of the disease progression and rapid improvement following IVIG therapy combined with conventional antibiotic therapy has been demonstrated in *M. pneumoniae* pneumonia. Moreover, the advantage of IVIG therapy in the early phase of the disease in patients unresponsive to corticosteroid and initial antibiotic therapy has been reported [31].

Studies highlight the advantage of systemic immune modulators like IVIG and corticosteroids in the acute phase of the ARDS. Reduced morbidity, inhibition of disease progression, and preventing permanent systemic organ injury are identified as outcomes of these treatments under sufficient dose and early administration [28]. On the contrary, other studies reported no beneficial effect in ARDS patients [32, 33], and the guideline of sepsis treatment has not suggested administration of IVIG [34]. Therefore, IVIG treatment in ARDS patients is still debatable.

## Experiences of using IVIG in the treatment of COVID-19

In the three cases reported by Cao et al*.* as well as the reported case of COVID-19 with mucous membrane pemphigoid (MMP) by Daneshpazhooh et al*.*, administration of IVIG improved the condition rapidly and showed its effect primarily by reducing patients’ fever. All the cases above were discharged in less than a week with considerable improvements [35, 36]. Furthermore, a case report by Shi et al. indicated that plasma exchange (PE) followed by IVIG provides clinical benefit for severe COVID-19 [37].

In line with these findings, Mohtadi et al. reported 5 cases of COVID-19, in whom administering hydroxychloroquine and antibacterial agents failed to improve the condition. Patients received IVIG at the dose of 0.3–0.5 g/kg for five days, in a way that no one received less than 25 g of IVIG. The clinical condition ameliorated markedly so that the patients were discharged from the hospital after that [38].

Another study reported a reduction in 28-day-mortality rates, the length of hospital stay, and the need for mechanical ventilation by IVIG treatment within 48 h after admission. However, IVIG administration in 24 h did not show this result. This highlights the relation between the time of IVIG administration and the mitigation of the mortality rate [39].

Furthermore, a multicenter retrospective cohort study by Shao et al. included 325 patients, from which 174 cases received IVIG. Consequently, only patients with severe COVID-19 from the IVIG group had better 28-day mortality rates. No effects were apparent on the total duration of the disease or the length of hospital stay [40]. A recent systematic review performed by Mansourabadi et al. suggested using IVIG therapy combined with antiviral drugs, according to the 63 cases of COVID-19 reported in the literature, who were successfully treated with IVIG [41].

The studies mentioned above have limitations that make it difficult to persuade the use of IVIG. Two of the main confounding factors are the small sample size and the simultaneous administration of other therapy methods (such as glucocorticoids and plasma exchange). To achieve a more accurate result, Gharebaghi et al. have recently performed a randomized placebo-controlled double-blind clinical trial with a total of 59 patients. They found out that administering 20 g/day of IVIG for three days reduces mortality, despite increasing hospital stay duration. Patients in the IVIG group survived and stayed longer in the hospital until full recovery, while patients in the control group died earlier. They suggested IVIG treatment in patients who were not improved with the antiviral and chloroquine-class drugs, whose oxygen saturation is persistently under 90%, patients with more than 30% of lung involvement in CT scan, and patients whose lungs are progressively being involved in serial CT scans [42].

There are also a few studies highlighting the effect of IVIG on neurologic complications associated with COVID-19. A case report by El-Zein et al. described a 40-year-old man with SARS-CoV-2-associated meningitis/encephalitis infection whose condition markedly improved with administration of IVIG for five days [43]. Muccioli et al. have performed a retrospective study on the data collected from patients developing encephalopathy who received IVIG at the dosage of 0.4 g/kg. Patients showed no adverse reactions to the therapy. All patients recovered completely after a mean time of 29.8 days. Improvements started to become evident in the first 3–4 days [44]. The recent case report from Freire-Alvarez et al. also showed clinical improvement upon the usage of IVIG and cytokine blocking drugs in an acute demyelinating encephalomyelitis associated with COVID-19 [45]. Considering the lack of clinical trials, it is difficult to conclude that IVIG usage upon COVID-19-related neurological manifestations is helpful.

## Immunologic mechanisms of using IVIG in COVID-19 patients

Patients commonly go through three clinical stages of the COVID-19: the acute or pneumonia stage, the viremia stage, and the recovery stage. The possible pathogenesis of COVID-19 is that lymphocyte count in the early phase of the disease (1–14 days) is normal or slightly low [41]. However, significant lymphopenia in the late phase (7–14 days after symptoms appear) occurs when viremia takes place. Furthermore, B lymphocyte reduction is observed during the early stage [2]. One of the diagnostic methods for COVID-19 is antibody measurement in patients’ serum. Most evidence indicates that a higher level of SARS-2-COVID-IgG is a marker of severe disease (although for unclear reasons at the moment) and antibody levels are more diagnostic than therapeutic markers [42–45].

IVIG treatment inhibits inflammatory mechanisms, including reduced production of IL-6 [22, 35], TNF‐α production, T-cell activation, matrix metalloproteinase nine activity [46], and IL-12/23p40 in macrophages [47]. On the other hand, anti-inflammatory mechanisms activate, for instance, the production of IL-10 in the gut and the macrophages and Peroxisome Proliferator-Activated Receptor gamma (PPARγ) increase. Simultaneously, the expression of Toll-like receptor-4 (TLR-4) is reduced [22, 35, 47]. It is determined that these effects of IVIG on the inflammatory storm do not incline patients towards immunosuppression [36]. Furthermore, IVIG may affect COVID-19 development through prevention of dendritic cell maturation, reduced IL-12 expression, and increased IL-33, IL-4, and IL-13 production [22, 46, 47].

## Challenges of using IVIG in COVID-19 patients

As mentioned earlier, several studies have shown the beneficial effects of using IVIG therapy against COVID-19 [36–38, 40–45, 48–50]. These studies are summarized in Table 1. Before describing the challenges of IVIG therapy, it is worth mentioning that not all the studies using IVIG as a treatment adjunct ended in positive results. Tables 2 and 3 illustrate the studies that reported no significant effect of IVIG and those that found inconclusive results.

**Table 1:** summary of the studies supporting the use of IVIG in COVID-19

Studies, that suggested the use of IVIG	Type of Study	Total Number of Cases	Dosage of IVIG	Measurement of treatment success	Other therapies used in the course of disease	Comorbidities	Additional explanations
Cao et al. [35]	Case series	3	25 g/day for 5 days or 0.3–0.4 g/kg/day for 5 days	Improvied clinical status (fever, O2 saturation and difficulty of breathing) after 5 days	Supportive care, oseltamvir, azithromycin, empirical moxifloxacin, lopinavir/ritonavir, prednisolone	Patient 1: previously generally healthy Patient 2: hypertension (2 years-well-controlled) Patient 3: previously generally healthy	–
Xie et al.[39]	Retrospective	58	20 g/day	28 day mortality rate	Moxifloxacin, low–molecular weight heparin (LMWH), hypoalbuminemia correction, Thymosin, glucocorticoids	–	Initiation of IVIG within the first 48 h of admission is beneficial
Shi et al.[37]	Case report	1	20 g for 3 days	Improved laboratory markers (such as lymphocyte count) and clinical status after 3 days	Supportive care, inhaled interferon alpha-2b, Lopinavir/Ritonavir, G-CSF, dopamine (vasopressor), empirical ceftriaxone, IV piperacillin/tazobactam thymalfasin, methylprednisolone, Plasma exchange (PE),	–	–
Daneshpazhooh et al. [36]	Case repost	1	2 g/kg total	Improved clinical symptoms and recovered lymphopenia	Prednisolone, rituximab, mycophenolate mofetil (MMF), hydroxychloroquine, oseltamivir, lopinavir/ritonavir, iv meropenem, vancomycin, ribavirin, levofloxacin,	Mucous Membrane Pemphigoid (MMP), diabetes, hypertension, benign prostatic hypertrophy	Minimizing or discontinuation of immunosuppressive medications is advised
Mohtadi et al.[38]	Case series	5	0.3–0.5 g/kg for 5 consecutive days, not less than 25 g for each patient	Improvement of pulmonary involvement in CT scan and O2 saturation after 5 days	hydroxychloroquine, Kaletra, oseltamivir, vancomycin, levofloxacin, Tavanxm Meropenem, ceftriaxone, azithromycin, imipenem hydrocortisone, Mycophenolic acid, Cyclosporine	Hypertension, kidney transplantation, diabetes, heart disease	–
Mansourabadi et al. [41]	Retrospective	80	0.3–0.5 g/kg/day for 5 days	Varied for different studies	convalescent plasma therapy, monoclonal antibodies, interferon, mesenchymal stem cell therapy, Tocilizumab, corticosteroid, and Hydroxychloroquine	–	–
El-Zein et al.[43]	Case Report	1	0.4 g/kg for 5 days	Improved neurological condition after 5 days	Hydroxychloroquine	–	–
Muccioli et al. [44]	Retrospective	5	0.4 g/kg for 19–55 days	Improvement of neurological symptoms in 3–4 days	Supportive care	Diabetes, hypertension, ischemic heart disease, iatrogenic parkinsonism, Bipolar disorder, Mild Cognitive Impairment, hypertensive cardiomyopathy	–
Gharebaghi et al.[42]	randomized placebo-controlled double-blind clinical trial	59	20 g/day of IVIG for three days	Lowered in hospital mortality rate in 7–9 days	Supportive care	–	–
Freire-Alvarez et al. [45]	Case report	1	0.4 g/kg for 5 days	Improved clinical condition and discontinuation of mechanical support after 5 days	Supportive care, Tocilizumab ( IL-6 receptor antagonist), intravenous acyclovir, lopinavir/ritonavir, subcutaneous interferon beta-1b	–	–
Sakoulas et al. [50]	Prospective Randomized Trial	33 (16 in the IVIG group)	0.5 g/kg/day for 3 days	Lowered length of hospital and ICU stay, lowered respiratory failures needed mechanical support, improved oxygenation	Methylprednisolone, remdesevir, convalescent plasma,	–	Lower rate of need for mechanical ventilation and a reduction in hospitalization length was observed in IVIG group

**Table 2:** summary of the studies reported no significant effect of IVIG administration in COVID-19

Studies suggesting against the use of IVIG	Type of Study	Number of Cases	Dosage of IVIG	Measurement of treatment success	Other therapies used in the course of disease	Comorbidities	Additional Explanations
Liu et al. [51]	Retrospective	109	–	Did not lower mortality rate in hospital stay (42 days)	Antivirals, glucocorticoid	Varied (diabetes, cerebrovascular disease, chronic kidney disease, etc.)	No significant effect of antivirus, glucocorticoid, or immunoglobulin treatment on survival was observed in patients with ARDS
Pei et al. [52]	Systematic review and meta-analysis	1142	Varied	Varied	Antivirals, antibiotics, glucocorticoid	Varied	intravenous immunoglobulin had a nonsignificant effect on mortality

**Table 3:** summary of the studies with inconclusive results about the use of IVIG in COVID-19

Studies with inconsistent results	Type of Study	Number of Cases	Dosage of IVIG	Measurement of treatment success	Other therapies used in the course of disease	Comorbidities	Additional Explanations
Shao et al. [40]	multicenter retrospective cohort	325	0.1 to 0.5 g/kg per day for 5 to 15 days	28 day mortality, 60 day mortality, length of hospital stay, length of disease period	Supportive care	Varied	IVIG therapy cannot reduce the total duration of the disease, only patients with severe COVID-19 from the IVIG group had better 28-day mortality rates
Tabarsi et al. [54]	Randomized control trial	84	400 mg/kg/day for three days	In hospital mortality rate, mechanical ventilation need, length of hospital stay	hydroxychloroquine, lopinavir/ritonavir and supportive care	Varied	No improvement in CT scan or the overall course of the disease was observed despite a reduction in the duration of hospitalization

Liu et al. found no notable impact on the patients with ARDS [51]. Furthermore, neither IVIG nor antibiotics brought about an increase in survival rate in Pei et al. study [52]. The systematic review performed by Zhang et al. found inconclusive results about the effectiveness of IVIG in COVID-19 [53]. Moreover, no improvement in CT scan or the overall course of the disease was observed in Tabarsi et al. study, despite a reduction in the duration of hospitalization [54]. Also, a review by Moradimajd et al. concluded that available literature is not enough to make the right decision about the efficacy of IVIG therapy in COVID-19 patients [55]. These studies provide a base for counterarguments in the effectiveness of IVIG therapy in SARS-CoV-2 infection. Therefore, more randomized clinical trials should be performed to shed light on the IVIG efficacy.

In addition to the controversies in using the IVIG, some other challenges need to be discussed. First of all, the life of many immunodeficient patients depends on the IVIG lots [56]. The COVID-19 pandemic inflicted additional pressure on the supply and demand balance, pushing it toward a shortage of IVIG [57]. Furthermore, the high price of IVIG (approximately $100/g) added to the infusion costs, make it a relatively expensive therapy [58].

Second, adverse effects of IVIG do not arise very often but happen in 10% of patients. In rare cases, thromboembolism happened to the patients upon receiving IVIG. Since COVID-19 itself is a hypercoagulable state [59], it is recommended to add anti-coagulation medications to the treatment and keep the patient hydrated [60, 61].

Third, using intravenous immunoglobulins can suppress the normal antibody response in the patients, leading to ineffective immunization against COVID-19. As a result, even if the patient recovers from the COVID-19, he/she may still be vulnerable and must be vaccinated as soon as possible [62].

Fourth, If the sera collected from the general population lacks the anti-SARS-CoV-2 antibodies, its use provides no benefit [63]. Sera derived from SARS patients [64] and sera before the pandemic [56] have no cross-neutralization with SARS-CoV-2 [25]. Conversely, Diez et al. claimed that the Gamunex®-C and Flebogamma® DIF (Grifols) intravenous immunoglobulin (IVIG) products, manufactured before the pandemic, cross-neutralize SARS, MERS, and the newly emerged SARS-CoV-2 [65]. The rationale behind this difference could be the dissimilarity of study methods, as Diez et al. [65] used antigens from EIA kits instead of virus-infected cells [66].

Some other risks should be considered too. Transferring of pathogens, serum sickness, transfusion-related acute lung injury (TRALI) [67, 68], and antibody-dependent enhancement (ADE). ADE requires prior exposure to similar antigens, suggesting that sera-containing antibodies against other coronaviruses may play a role in this phenomenon. But, it is still debatable that whether the IVIG can promote ADE or not [62, 69, 70].

In conclusion, it is still difficult to determine whether or not IVIG should be included in the treatment strategy of COVID-19 patients. More randomized clinical trials with large sample sizes are required to decide the amount of IVIG efficacy.

## Conclusion

This study does not recommend IVIG therapy as a definitive cure for the acute phase of COVID-19 but instead summarizes the evidence and gives clues for upcoming trials. Several studies reported the advantage of IVIG therapy for COVID-19 patients. However, these studies are primarily case reports with small sample sizes and uncontrolled confounding factors. These uncertainties and the shortage of IVIG supply and its high cost suggest that its use must be supported with strong evidence. Therefore, more clinical trials on the effect of IVIG therapy in COVID-19 patients should be performed to establish a certain conclusion about IVIG effectiveness.

## Data Availability

Data of patient is available, if needed.

## Abbreviations

ADCC: Antibody-dependent cellular cytotoxicity
ADE: Antibody- dependent Enhancement
APC: Antigen-presenting cells
ARDS: Acute respiratory distress syndrome
CRP: C-reactive protein
COVID-19: Coronavirus disease
ECMO: Extracorporeal membrane oxygenation
FcγRs: Fcγ receptors
FcRn: Fc receptor
GM-CSF: Granulocyte–macrophage colony-stimulating factor
IFN-γ: Interferon-gamma
IL: Interleukin
IP-10: Inducible protein 10
IVIG: Intravenous immunoglobulin
LDH: Lactate dehydrogenase
LMWH: Low molecular weight heparin
MERS: Middle East respiratory syndrome
MMP: Membrane pemphigoid
SARS: Severe acute respiratory syndrome
SARS-CoV-2: Severe acute respiratory syndrome coronavirus 2
TNF-α: Tumor necrosis factor-alpha

## References

[1] Hanaei S, Rezaei N (2020). COVID-19: Developing from an outbreak to a pandemic. Arch Med Res.

[2] WHO. Estimating Mortality from COVID-19. https://www.who.int/news-room/commentaries/detail/estimating-mortality-from-covid-19. Accessed 27 Sept 2021.

[3] Petersen E, Koopmans M, Go U, Hamer DH, Petrosillo N, Castelli F (2020). Comparing SARS-CoV-2 with SARS-CoV and influenza pandemics. Lancet Infect Dis.

[4] Kaur SP, Gupta V (2020). COVID-19 vaccine: a comprehensive status report. Virus Res.

[5] Huang C, Wang Y, Li X, Ren L, Zhao J, Hu Y (2020). Clinical features of patients infected with 2019 novel coronavirus in Wuhan, China. Lancet.

[6] Yazdanpanah F, Hamblin MR, Rezaei N (2020). The immune system and COVID-19: friend or foe?. Life Sci.

[7] Zhang C, Wu Z, Li JW, Zhao H, Wang GQ (2020). The cytokine release syndrome (CRS) of severe COVID-19 and Interleukin-6 receptor (IL-6R) antagonist Tocilizumab may be the key to reduce the mortality. Int J Antimicrob Agents..

[8] Saghazadeh A, Rezaei N (2020). Immune-epidemiological parameters of the novel coronavirus—a perspective. Expert Rev Clin Immunol..

[9] Zhou P, Yang XL, Wang XG, Hu B, Zhang L, Zhang W (2020). A pneumonia outbreak associated with a new coronavirus of probable bat origin. Nature.

[10] Eibl MM (2008). History of immunoglobulin replacement. Immunol Allergy Clin North Am..

[11] Galeotti C, Kaveri SV, Bayry J (2017). IVIG-mediated effector functions in autoimmune and inflammatory diseases. Int Immunol.

[12] Wunderink RG (2017). Other community respiratory viruses. Clin Chest Med.

[13] Rezaei N, Abolhassani H, Aghamohammadi A, Ochs HD (2011). Indications and safety of intravenous and subcutaneous immunoglobulin therapy. Expert Rev Clin Immunol.

[14] Kaveri SV, Maddur MS, Hegde P, Lacroix-Desmazes S, Bayry J (2011). Intravenous immunoglobulins in immunodeficiencies: more than mere replacement therapy. Clin Exp Immunol..

[15] Bayry J, Fournier EM, Maddur MS, Vani J, Wootla B, Sibéril S (2011). Intravenous immunoglobulin induces proliferation and immunoglobulin synthesis from B cells of patients with common variable immunodeficiency: a mechanism underlying the beneficial effect of IVIg in primary immunodeficiencies. J Autoimmun.

[16] Trinath J, Hegde P, Sharma M, Maddur MS, Rabin M, Vallat JM (2013). Intravenous immunoglobulin expands regulatory T cells via induction of cyclooxygenase-2-dependent prostaglandin E2 in human dendritic cells. Blood.

[17] Maddur MS, Rabin M, Hegde P, Bolgert F, Guy M, Vallat JM (2014). Intravenous immunoglobulin exerts reciprocal regulation of Th1/Th17 cells and regulatory T cells in Guillain-Barré syndrome patients. Immunol Res.

[18] Janke AD, Yong VW (2006). Impact of IVIg on the interaction between activated T cells and microglia. Neurol Res.

[19] Hung IFN, To KKW, Lee CK, Lee KL, Yan WW, Chan K (2013). Hyperimmune IV immunoglobulin treatment: a multicenter double-blind randomized controlled trial for patients with severe 2009 influenza A(H1N1) infection. Chest.

[20] Diebel LN, Liberati DM, Diglio CA, Brown WJ (2005). Immunoglobulin A modulates inflammatory responses in an in vitro model of pneumonia. J Trauma Acute Care Surg.

[21] Busse PJ, Razvi S, Cunningham-Rundles C (2002). Efficacy of intravenous immunoglobulin in the prevention of pneumonia in patients with common variable immunodeficiency. J Allergy Clin Immunol.

[22] Jawhara S (2020). Could intravenous immunoglobulin collected from recovered coronavirus patients protect against COVID-19 and strengthen the immune system of new patients?. Int J Mol Sci..

[23] Mair-Jenkins J, Saavedra-Campos M, Baillie JK, Cleary P, Khaw FM, Lim WS (2015). The effectiveness of convalescent plasma and hyperimmune immunoglobulin for the treatment of severe acute respiratory infections of viral etiology: a systematic review and exploratory meta-analysis. J Infect Dis.

[24] Stockman LJ, Bellamy R, Garner P (2006). SARS: systematic review of treatment effects. PLoS Med..

[25] Kubota-Koketsu R, Terada Y, Yunoki M, Sasaki T, Nakayama EE, Kamitani W (2020). Neutralizing and binding activities against SARS-CoV-1/2, MERS-CoV, and human coronaviruses 229E and OC43 by normal human intravenous immunoglobulin derived from healthy donors in Japan. Transfusion.

[26] Luke T, Wu H, Zhao J, Channappanavar R, Coleman CM, Jiao JA (2016). Human polyclonal immunoglobulin G from transchromosomic bovines inhibits MERS-CoV in vivo. Sci Transl Med..

[27] Mustafa S, Balkhy H, Gabere MN (2018). Current treatment options and the role of peptides as potential therapeutic components for Middle East Respiratory Syndrome (MERS): a review. J Infect Public Health.

[28] Lee KY (2017). Pneumonia, acute respiratory distress syndrome, and early immune-modulator therapy. Int J Mol Sci..

[29] Liu Q, Zhou YH, Yang ZQ (2016). The cytokine storm of severe influenza and development of immunomodulatory therapy. Cell Mol Immunol.

[30] Hui DS, Lee N, Chan PK, Beigel JH (2018). The role of adjuvant immunomodulatory agents for treatment of severe influenza. Antiviral Res.

[31] Youn YS, Lee SC, Rhim JW, Shin MS, Kang JH, Lee KY (2014). Early additional immune-modulators for mycoplasma pneumoniae pneumonia in children: an observation study. Infect Chemother.

[32] Brocklehurst P, Farrell B, King A, Juszczak E, Darlow B, Haque K (2011). Treatment of neonatal sepsis with intravenous immune globulin. N Engl J Med.

[33] Prohaska S, Schirner A, Bashota A, Körner A, Blumenstock G, Haeberle HA (2018). Intravenous immunoglobulin fails to improve ARDS in patients undergoing ECMO therapy. J Intensive Care.

[34] Rhodes A, Evans LE, Alhazzani W, Levy MM, Antonelli M, Ferrer R (2017). Surviving sepsis campaign: international guidelines for management of sepsis and septic shock: 2016. Crit Care Med.

[35] Cao W, Liu X, Bai T, Fan H, Hong K, Song H (2020). High-dose intravenous immunoglobulin as a therapeutic option for deteriorating patients with coronavirus disease 2019. Open Forum Infect Dis.

[36] Daneshpazhooh M, Soori T, Isazade A, Noormohammadpour P (2020). Mucous membrane pemphigoid and COVID-19 treated with high-dose intravenous immunoglobulins: a case report. J Dermatolog Treat.

[37] Shi H, Zhou C, He P, Huang S, Duan Y, Wang X (2020). Successful treatment of plasma exchange followed by intravenous immunogloblin in a critically ill patient with 2019 novel coronavirus infection. Int J Antimicrob Agents..

[38] Mohtadi N, Ghaysouri A, Shirazi S, Sara A, Shafiee E, Bastani E (2020). Recovery of severely ill COVID-19 patients by intravenous immunoglobulin (IVIG) treatment: a case series. Virology.

[39] Xie Y, Cao S, Dong H, Li Q, Chen E, Zhang W (2020). Effect of regular intravenous immunoglobulin therapy on prognosis of severe pneumonia in patients with COVID-19. J Infect.

[40] Shao Z, Feng Y, Zhong L, Xie Q, Lei M, Liu Z (2020). Clinical efficacy of intravenous immunoglobulin therapy in critical ill patients with COVID-19: a multicenter retrospective cohort study. Clin Transl Immunol..

[41] Mansourabadi AH, Sadeghalvad M, Mohammadi-Motlagh HR, Rezaei N (2020). The immune system as a target for therapy of SARS-CoV-2: a systematic review of the current immunotherapies for COVID-19. Life Sci.

[42] Gharebaghi N, Nejadrahim R, Mousavi SJ, Sadat-Ebrahimi SR, Hajizadeh R (2020). The use of intravenous immunoglobulin gamma for the treatment of severe coronavirus disease 2019: a randomized placebo-controlled double-blind clinical trial. BMC Infect Dis.

[43] El-Zein RS, Cardinali S, Murphy C, Keeling T (2020). COVID-19-associated meningoencephalitis treated with intravenous immunoglobulin. BMJ Case Rep.

[44] Muccioli L, Pensato U, Bernabè G, Ferri L, Tappatà M, Volpi L (2020). Intravenous immunoglobulin therapy in COVID-19-related encephalopathy. J Neurol.

[45] Freire-Álvarez E, Guillén L, Lambert K, Baidez A, García-Quesada M, Andreo M (2020). COVID-19-associated encephalitis successfully treated with combination therapy. Clin Infect Pract..

[46] Prete M, Favoino E, Catacchio G, Racanelli V, Perosa F (2020). SARS-CoV-2 infection complicated by inflammatory syndrome. Could high-dose human immunoglobulin for intravenous use (IVIG) be beneficial?. Autoimmun Rev..

[47] Rojas M, Rodríguez Y, Monsalve DM, Acosta-Ampudia Y, Camacho B, Gallo JE (2020). Convalescent plasma in Covid-19: possible mechanisms of action. Autoimmun Rev..

[48] Aljaberi R, Wishah K (2020). Positive outcome in a patient with coronavirus disease 2019 and common variable immunodeficiency after intravenous immunoglobulin. Ann Allergy Asthma Immunol.

[49] Quinti I, Lougaris V, Milito C, Cinetto F, Pecoraro A, Mezzaroma I (2020). A possible role for B cells in COVID-19? Lesson from patients with agammaglobulinemia. J Allergy Clin Immunol.

[50] Sakoulas G, Geriak M, Kullar R, Greenwood K, Habib M, Vyas A (2020). Intravenous immunoglobulin (IVIG) significantly reduces respiratory morbidity in COVID-19 pneumonia: a prospective randomized trial. MedRxiv..

[51] Liu Y, Sun W, Li J, Chen L, Wang Y, Zhang L (2020). Clinical features and progression of acute respiratory distress syndrome in coronavirus disease 2019. MedRxiv..

[52] Pei L, Zhang S, Huang L, Geng X, Ma L, Jiang W (2020). Antiviral agents, glucocorticoids, antibiotics, and intravenous immunoglobulin in 1142 patients with coronavirus disease 2019: a systematic review and meta-analysis. Pol Arch Intern Med.

[53] Zhang L, Liu Y (2020). Potential interventions for novel coronavirus in China: a systematic review. J Med Virol.

[54] Tabarsi P, Barati S, Jamaati H, Haseli S, Marjani M, Moniri A (2020). Evaluating the effects of Intravenous Immunoglobulin (IVIg) on the management of severe COVID-19 cases: A randomized controlled trial. Int Immunopharmacol..

[55] Moradimajd P, Samaee H, Sedigh-Maroufi S, Kourosh-Aami M, Mohsenzadagan M (2021). Administration of intravenous immunoglobulin in the treatment of COVID-19: A review of available evidence. J Med Virol.

[56] Schwaiger J, Karbiener M, Aberham C, Farcet MR, Kreil TR (2020). No SARS-CoV-2 neutralization by intravenous immunoglobulins produced from plasma collected before the 2020 pandemic. J Infect Dis.

[57] Hartmann J, Klein HG (2020). Supply and demand for plasma-derived medicinal products—a critical reassessment amid the COVID-19 pandemic. Transfusion.

[58] Stiehm ER (2013). Adverse effects of human immunoglobulin therapy. Transfus Med Rev.

[59] Lünemann JD, Nimmerjahn F, Dalakas MC (2015). Intravenous immunoglobulin in neurology–mode of action and clinical efficacy. Nat Rev Neurol.

[60] Huang L, Kanellis J, Mulley W (2011). Slow and steady. Reducing thrombotic events in renal transplant recipients treated with IVIg for antibody-mediated rejection. Nephrology (Carlton).

[61] Soy M, Keser G, Atagündüz P, Tabak F, Atagündüz I, Kayhan S (2020). Cytokine storm in COVID-19: pathogenesis and overview of anti-inflammatory agents used in treatment. Clin Rheumatol.

[62] Pourahmad R, Moazzami B, Rezaei N (2020). Efficacy of Plasmapheresis and Immunoglobulin Replacement Therapy (IVIG) on Patients with COVID-19. SN Compr Clin Med..

[63] Alhazzani W, Møller MH, Arabi YM, Loeb M, Gong MN, Fan E (2020). Surviving Sepsis Campaign: guidelines on the management of critically ill adults with Coronavirus Disease 2019 (COVID-19). Intensive Care Med.

[64] Anderson DE, Tan CW, Chia WN, Young BE, Linster M, Low JH (2020). Lack of cross-neutralization by SARS patient sera towards SARS-CoV-2. Emerg Microbes Infect.

[65] Díez J-M, Romero C, Gajardo R (2020). Currently available intravenous immunoglobulin contains antibodies reacting against severe acute respiratory syndrome coronavirus 2 antigens. Immunotherapy.

[66] Kubota-Koketsu R, Terada Y, Yunoki M, Sasaki T, Nakayama EE, Kamitani W (2020). Neutralizing and binding activities against SARS-CoV-1/2, MERS-CoV, and human coronaviruses 229E and OC43 by normal human intravenous immunoglobulin derived from healthy donors in Japan. Transfusion.

[67] Alijotas-Reig J, Esteve-Valverde E, Belizna C, Selva-O'Callaghan A, Pardos-Gea J, Quintana A (2020). Immunomodulatory therapy for the management of severe COVID-19. Beyond the anti-viral therapy: a comprehensive review. Autoimmun Rev..

[68] Vlaar AP, Juffermans NP (2013). Transfusion-related acute lung injury: a clinical review. Lancet.

[69] Tetro JA (2020). Is COVID-19 receiving ADE from other coronaviruses?. Microbes Infect.

[70] Nguyen AA, Habiballah SB, Platt CD, Geha RS, Chou JS, McDonald DR (2020). Immunoglobulins in the treatment of COVID-19 infection: Proceed with caution. Clin Immunol..

